# Dose, duration and strain of bacillus Calmette–Guerin in the treatment of nonmuscle invasive bladder cancer: Meta-analysis of randomized clinical trials

**DOI:** 10.1097/MD.0000000000008300

**Published:** 2017-10-20

**Authors:** Yongjun Quan, Chang Wook Jeong, Cheol Kwak, Hyeon Hoe Kim, Hyung Suk Kim, Ja Hyeon Ku

**Affiliations:** a Department of Urology, Seoul National University Hospital, Seoul; b Department of Urology, Dongguk University Ilsan Medical Center, Goyang, Korea.

**Keywords:** BCG vaccine, carcinoma, disease progression, recurrence, transitional cell, urinary bladder neoplasms

## Abstract

**Background:**

Intravesical bacillus Calmette–Guerin (BCG) instillation is widely used as an adjuvant therapy after transurethral resection of bladder tumor (TURBT) in patients with intermediate- and high-risk nonmuscle invasive bladder cancer (NMIBC). However, the effective dose, duration, and strain of BCG have not yet been clearly determined. We aimed to elucidate the relationship between dose, duration, and strain of BCG and clinical outcomes in NMIBC patients treated with TURBT.

**Methods:**

We conducted a literature search in Embase, Scopus, and PubMed databases for all relevant articles published up to October 2016 in accordance with the Preferred Reporting Items for Systematic Review and Meta-analysis guidelines. The relative risks of clinical outcomes, including recurrence, progression, cancer-specific mortality, and all-cause mortality according to dose (standard vs low), duration (induction vs maintenance), and strain of BCG were presented as the pooled risk ratio (RR) and 95% confidence interval (CI).

**Results:**

Nineteen studies meeting the inclusion criteria were finally selected in this meta-analysis. The risk of recurrence was significantly highly observed in case of low-dose BCG (RR, 1.17; 95% CI 1.06–1.30) and induction BCG (RR, 1.33; 95% CI 1.17–1.50) only group without heterogeneity among the included studies. Although there were no significant differences between dose or duration and other clinical outcomes. On direct comparison in each study comparing BCG strains, the Tice stain showed a relatively high probability of recurrence compared with the Connaught (RR, 1.29; 95% CI 1.01–1.64) and RIVM (RR, 2.04, 95% CI 1.28–3.25) strains. Funnel plot testing revealed no significant publication bias.

**Conclusion:**

The use of standard dose and maintenance BCG instillation may be effective to reduce recurrence rate after TURBT for NMIBC. Further large scale, well-designed, and prospective studies, with stratification of the patients into risk group at randomization, will be required to determine the optimal guideline of BCG use to improve clinical outcomes in NMIBC.

## Abstract 2

Supplemental Digital Content is available in the text

## 1 Introduction

Nonmuscle invasive bladder cancer (NMIBC), which accounts for approximately 75% of initially diagnosed bladder cancers and consists of either tumor confined to the mucosa (Ta, carcinoma in situ [CIS]) or tumor invading the submucosa (T1), is primarily treated with transurethral resection of bladder tumor (TURBT). NMIBC has considerably varied clinical behaviors depending on the risk of disease recurrence and progression after TURBT.^[1,2]^ Depending on the European Organization of Research and Treatment of Cancer risk tables,^[3,4]^ low-risk tumors (<3 cm, solitary, Ta, low grade [LG]) have an average of recurrence rate of 20% (15%–31%), but show a low progression rate to muscle invasive disease of less than 1%. In contrast, intermediate- (multiple and/or ≥3 cm, Ta, LG) and high-risk tumors (T1 and/or high grade (HG) and/or CIS) have high recurrence rates ranging from 24% to 78% and high potential risk to progress into muscle invasive disease (1%–45%).

A crucial issue in the management of NMIBC is the reduction of disease recurrence and prevention of progression into muscle invasive disease. Progression leads to the need of adjuvant therapy in almost all NMIBC patients treated with TURBT. Intravesical bacillus Calmette–Guerin (BCG) instillation has been widely used as the mainstay of adjuvant therapy after TURBT in NMIBC patients.^[5]^ The efficacy and safety of adjuvant BCG immunotherapy for the treatment of NMIBC have been proven by several randomized controlled trials (RCTs) and meta-analyses.^[6–10]^ Current international guidelines recommend using intravesical BCG instillation in intermediate- and high-risk NMIBC patients to decrease the risk of disease recurrence and progression.^[1,2,11]^

However, the effective dose, duration, and strain of BCG for intravesical instillation have not yet been clearly determined. Although a number of RCTs have assessed the differences of clinical outcomes according to dose (standard vs low),^[12–19]^ duration (induction vs maintenance),^[20–27]^ and strain of BCG^[28–30]^ in NMIBC, conflicting results have prevented any consensus concerning the effective BCG strategy.

In the present study, we sought to evaluate whether the clinical outcomes show significant difference according to dose, duration, and strain of used BCG in NMIBC through a systematic review and meta-analysis of relevant published RCTs.

## 2 Materials and methods

### 2.1 Ethics statement

Ethical approval or informed consent was not necessary for this meta-analysis because our analysis has not affected participants directly, and required data were extracted from previous published studies.

### 2.2 Search strategy

We conducted the current study according to Cochrane Collaboration and Preferred Reporting Items for Systematic Review and Meta-analysis guidelines.^[31]^ A comprehensive literature search was made using the Embase, Scopus, and PubMed databases. All articles in English published up to October 31, 2016 identified using the following search terms were used as key words separately or in combination were identified: “bladder cancer,” “BCG,” and “randomized.” Citation lists of all retrieved studies were then used to identify other potentially relevant publications. Two reviewers (YQ and CWJ) independently selected the relevant articles, and any conflicts between reviewers reached a consensus after discussion.

### 2.3 Inclusion and exclusion criteria

Depending on the Preferred Reporting Items for Systematic Review and Meta-analysis guidelines, we adopted the population, intervention, comparator, outcome, and study design approach to define study eligibility.^[31]^ The population was patients with NMIBC. The intervention was intravesical BCG immunotherapy. The comparator was dose, duration, and strain of BCG. The outcome was recurrence-free survival (RFS), progression-free survival (PFS), cancer-specific survival (CSS), and overall survival (OS). The study design was a meta-analysis of RCTs. Studies were considered eligible for further evaluation if they met the following inclusion criteria: original article; human research; English language; histologically conformed NMIBC; primarily treated with TURBT; availability of Kaplan–Meier/uni- or multivariable Cox proportional hazard models-derived results describing the differences of outcomes depending on dose, duration, and strain of BCG; and RCTs. The exclusion criteria were: letters, commentaries, case reports, reviews, and conference abstracts owing to limited data; articles in other languages than English; studies using other analyses instead of survival analysis; and overlapping articles or those with duplicated data. If the same study subjects or analyses of repeat data were found in more than 1 publication, only the most recent or the largest study was preferentially included in the analysis to avoid duplication of the same survival data. Each study was screened by 2 independent reviewers (CK and HHK) according to study eligibility. Any disagreements were resolved by consensus through discussion.

The primary endpoint of the meta-analysis was RFS. Recurrence was defined as any tumor relapse, either local or systemic, irrespective of bladder muscle invasion after TURBT. Secondary endpoints included PFS, CSS, and OS. Progression was defined as either increase of pathologic tumor stage and/or grade or the emergence of bladder muscle invasive disease with or without distant metastasis. CSS and OS were defined as the interval from the time of TURBT to death from bladder cancer and/or any cause, respectively.

### 2.4 Data extraction

Three investigators (YQ, HSK, and JHK) independently reviewed each eligible article and retrieved data from all publications meeting the inclusion criteria. Retrieved data were subsequently crosschecked to ensure their accuracy and any disparities among investigators reached a consensus after discussion. Information was extracted according to the reporting recommendations for tumor marker prognostic studies guidelines for reporting prognostic marker^[32]^ including: publication data (name of first author, publication year, geographic location, and recruitment period), characteristics of the study population (sample size including randomized number and eligible population, median age with range, gender, definition of progression, and median follow-up (FU) duration with range), tumor characteristics (tumor stage and grade) in each study, treatment characteristics in each study (dose, duration, and strain of BCG regimen used in each randomized group), and statistical data (survival curves, exact data of total, and exposed number in case and control groups) as well as hazard ratios (HRs) and their confidence intervals (CIs). Discrepancies were discussed to reach consensus.

### 2.5 Statistical analyses

The meta-analysis used the DerSimonian and Laird random effects model,^[33]^ applying the inverse of variance as a weighing factor, which provided the pooled risk ratios (RRs) with 95% CIs suggesting the difference of survival outcomes depending on each BCG regimen. For each trial, survival data were extracted and estimated according to previously described methods.^[34]^ If RRs and the corresponding 95% CIs were not directly reported, previously reported indirect methods were used to extract the log HR and variance.^[35]^ To evaluate the interstudy heterogeneity for the pooled RRs, we adopted both the Chi-square-based Q statistic and Higgins I-squared statistic test,^[36]^ which demonstrates the percentage of total variation among studies caused by heterogeneity rather than by chance. We judged that *P* < .05 for the Q test or an *I*^2^ statistic >50% implied the presence of significant heterogeneity across selected studies. Publication bias was assessed using the funnel plot. A symmetrical inspection of inverted funnel was regarded as no significant publication bias. In contrast, in case of the presence of bias, the inverted funnel plot should appear skewed and asymmetrical. All the *P*-values and 95% CIs were 2-sided, and *P* < .05 was considered statistically significant. The meta-analysis was conducted using Version 5.0 RevMan statistical software (Cochrane Collaboration, Copenhagen, Denmark).

## 3 Results

### 3.1 Study selection

**Figure 1:**
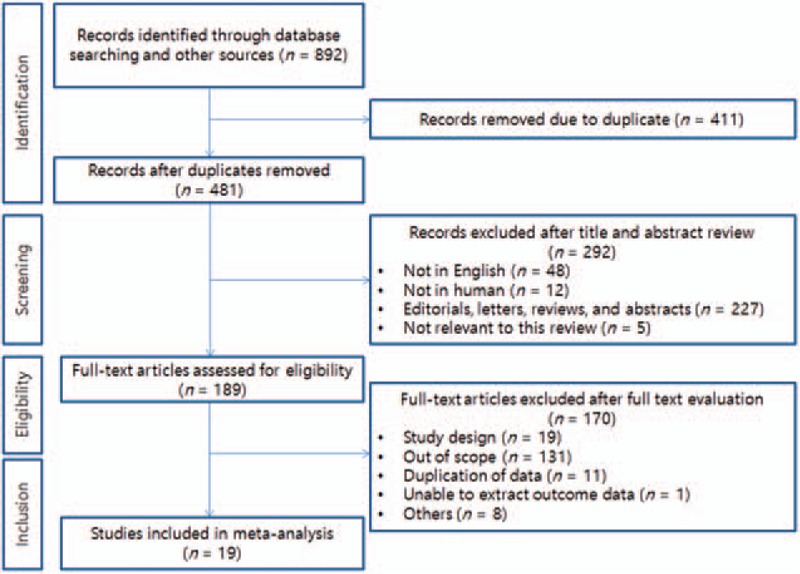
Flow chart of the literature search approach.

The initial database search identified 892 articles. Among these, 733 articles were excluded: 441 were duplicate publications and 292 articles were excluded after reviewing the corresponding titles and abstracts. A total of 189 articles remained for full text evaluation. Further review excluded 131 articles because they were irrelevant to the current analysis, 19, because the study design was not an RCT; 11, because data were overlapped with another study; and 9, owing to other causes. Finally, 19 articles were included for the meta-analysis.^[12–30]^Figure 1 shows a flow diagram of the selection process for relevant studies.

### 3.2 Study characteristics

The study and treatment characteristics of the 19 eligible studies are summarized in Tables 1 and 2. All the studies were prospective RCTs. These eligible studies were published from 1987 to 2016. Among studies, 9 were performed in Europe,^[13,14,16,19,21,23,26,28,30]^ 7 in Asia,^[15,17,18,24,25,27,29]^ and 3 in America.^[12,20,22]^ The median FU duration ranged from 2.7 to 120 months, while 6 studies did not suggest clear median FU duration. The pathologic tumor stage in all trials consisted of nonmuscle invasive urothelial carcinoma, including T1 and/or HG (grade 3) and/or CIS. The number of the studies comparing dose, duration, and strain of BCG were eight,^[12–19]^ eight,^[20–27]^ and three,^[28–30]^ respectively. In case of studies comparing BCG dose, the definition of low and standard dose showed some variations among studies. Either 80, 81, or 120 mg was used as a standard dose in the most studies, and the low dose was defined as a half or one/two-third of the standard dose in the most studies.^[12–18]^ One study did not clearly provide an exact BCG dose, but compared the efficacy between one-third and full-dose BCG at 1 and 3 years.^[19]^ Therefore, we pooled the 2 studies having different duration as each separate study comparing BCG dose when performing the meta-analysis. The final number of studies comparing BCG dose was considered as 9 (Table 2). On the studies comparing duration of BCG, the regimen of induction BCG therapy was identical as once weekly for 6 weeks in most studies. Only 1 trial used BCG once weekly for 8 weeks for the purpose of induction.^[24]^ Although the regimen of maintenance BCG was variably adopted among studies. The studies comparing the BCG strain (Oncotice, RIVM, ToKyo 172, and Connaught) were conducted under the conditions of induction BCG therapy only.

**Table 1:**
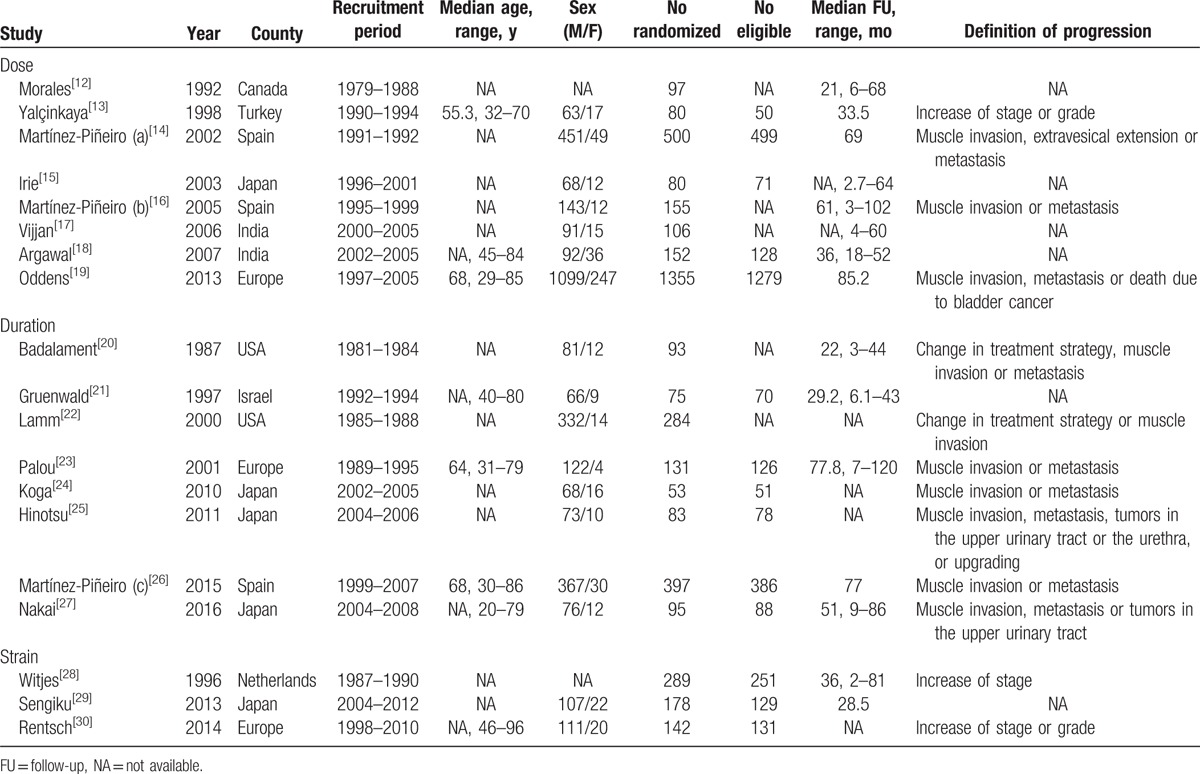
Randomized trials comparing doses, durations, and strains of bacillus Calmette–Guerin.

**Table 2:**
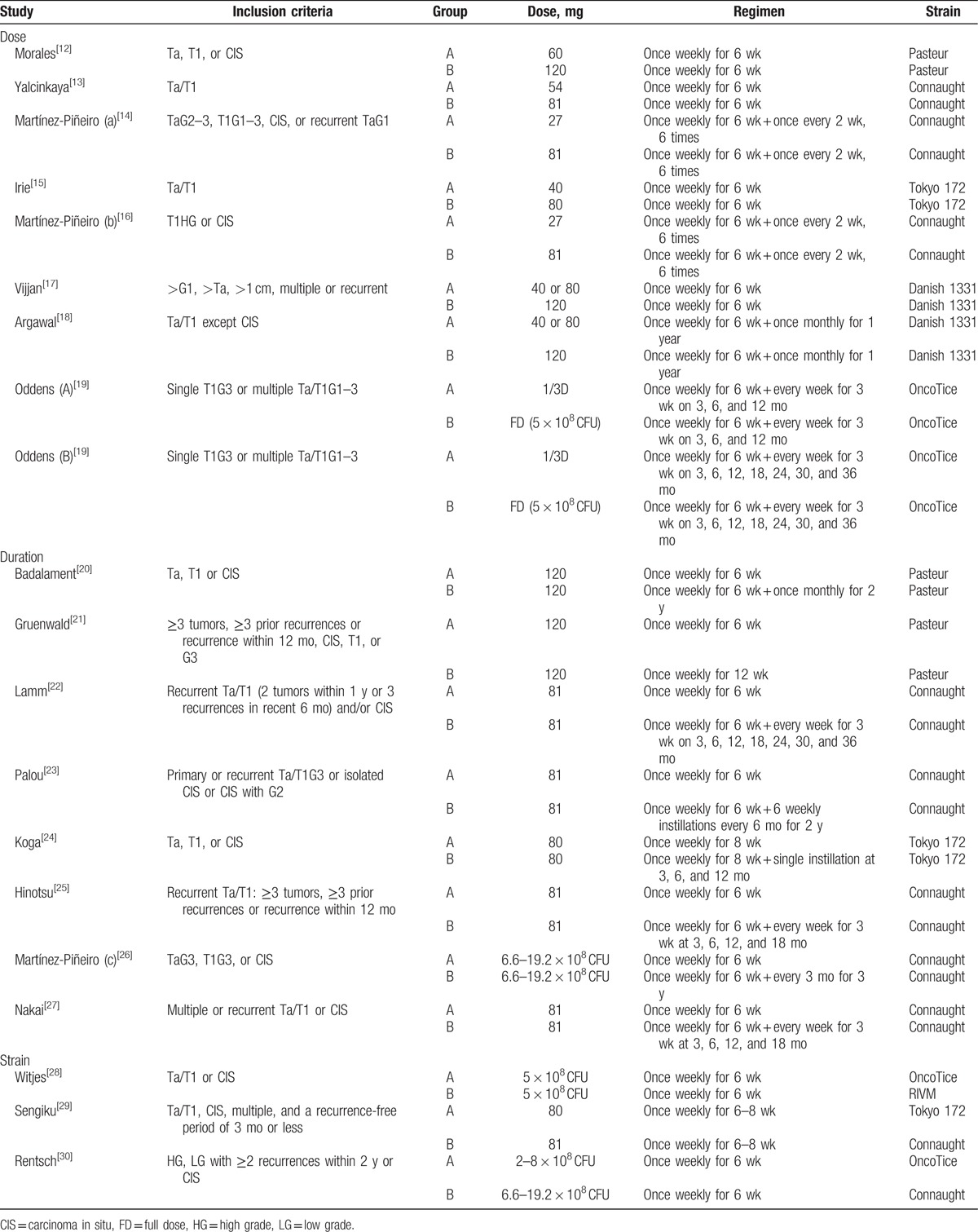
Treatment characteristics of the eligible studies.

### 3.3 Meta-analysis

#### 3.3.1 Dose

**Figure 2:**
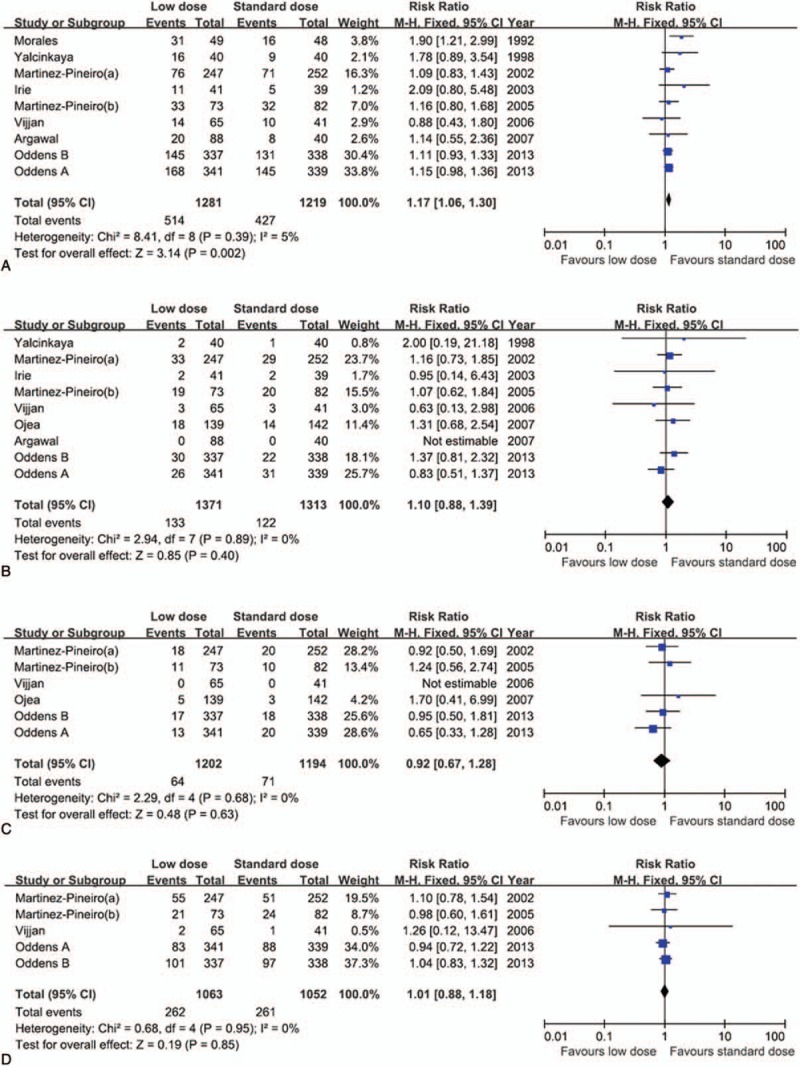
Forest plots of the prognosis of bacillus Calmette–Guerin (BCG) dose. The horizontal lines correspond to the study-specific hazard ratio and 95% confidence interval, respectively. The area of the squares reflects the study-specific weight. The diamond represents the results for pooled hazard ratio and 95% confidence interval. (A) recurrence-free survival, (B) progression-free survival, (C) cancer-specific survival, and (D) overall survival.

The pooled analysis of RFS was based on 9 studies. Compared with standard dose BCG, low-dose BCG was significantly related to worse RFS (RR, 1.17; 95% CI, 1.06–1.30). There was no obvious heterogeneity (*P* = .39; *I*^2^ = 5%; Fig. 2A). Significant differences were not found in PFS, CSS, and OS between low and standard BCG dose, and there was no interstudy heterogeneity in all analyses (Fig. 2B–D).

#### 3.3.2 Duration

**Figure 3:**
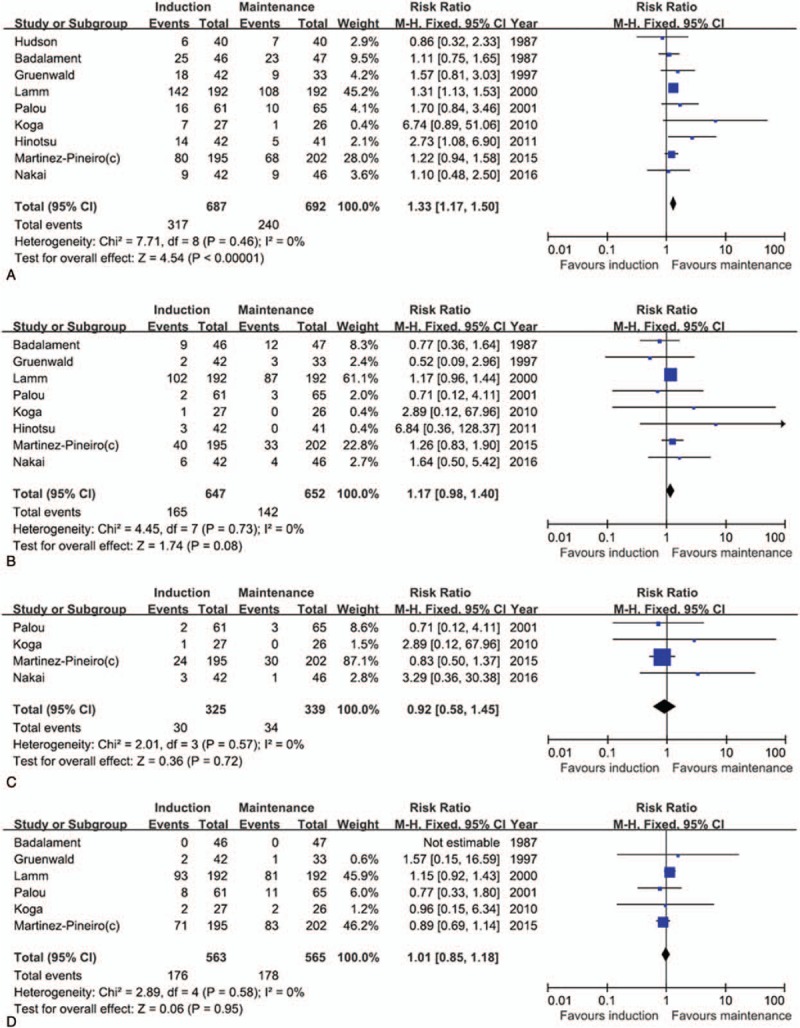
Forest plots of the prognosis of bacillus Calmette–Guerin (BCG) duration. The horizontal lines correspond to the study-specific hazard ratio and 95% confidence interval, respectively. The area of the squares reflects the study-specific weight. The diamond represents the results for pooled hazard ratio and 95% confidence interval. (A) recurrence-free survival, (B) progression-free survival, (C) cancer-specific survival, and (D) overall survival.

A total of 8 studies were included in the meta-analysis of RFS. Compared to maintenance BCG, induction BCG significantly showed a worse RFS (RR, 1.33; 95% CI, 1.17–1.50). The result for the test for heterogeneity was not significant (*P* = .46; *I*^2^ = 0%; Fig. 3A). In contrast, in the meta-analyses of the correlation between BCG duration and secondary endpoints (PFS, CSS, and OS), there were no significant differences according to BCG regimen (induction vs maintenance), and significant interheterogeneity was not observed in the analyses (Fig. 3B–D).

#### 3.3.3 Strain

Owing to the small number of studies included,^[28–30]^ meta-analysis was not performed with regard to the strains of BCG. Instead, when conducting direct comparison according to BCG strain in each study, the OncoTice strain (vs Connaught [RR, 1.29; 95% CI 1.01–1.64] or vs RIVM [RR 2.04; 95% CI 1.28–3.25]) was more likely to show a worse recurrence in some studies^[28,30]^ (supplemental Table S1). However, there were no meaningful correlations between BCG strain and other survival outcomes (RFS, CSS, and OS) (supplemental Table S1).

### 3.4 Publication bias

No significant publication bias was found in the meta-analyses of all survival outcomes according to various BCG regimens. Funnel plots for publication bias of the correlation between various BCG regimens (dose and duration) and survival outcomes (RFS, PFS, CSS, and OS) demonstrated a certain degree of asymmetry (supplemental Fig. S1A–D, and Fig. S2A–D).

## 4 Discussion

BCG is an attenuated mycobacterium developed as a vaccine for tuberculosis. It has shown an antitumor effect in several different cancers including bladder cancer. Intravesical BCG is widely used and has been one of the most successful immunotherapies for the management of NMIBC by inducing massive local immune response within the bladder.^[5]^ The preventive effect of BCG on tumor recurrence and progression in NMIBC has already been proven by several investigators.^[6,7,9]^ Therefore, based on risk predicting models, such as the European Organization of Research and Treatment of Cancer risk tables and Spanish Urological Club for Oncological Treatment scoring system,^[3,4,37–39]^ for recurrence and progression after TURBT for NMIBC, the international guidelines have recommended the use of intavesical BCG as an adjuvant therapy in intermediate-to-high risk NMIBC cases to remove the residual tumor and prevent recurrence and progression.^[1,2,11]^

However, the optimal treatment dose, duration, and strain of BCG have not yet been definitely established. Although there have been a number of prospective trials assessing the optimal duration and dose of BCG in NMIBC, the conflicting results have been reported among studies.

As for BCG dose, 1 prospective RCT comparing standard (81 mg) versus low (54 mg) dose demonstrated that recurrence rates significantly differed (0.71/month in standard vs 1.49/month in low; *P* < .05) but there were no significant differences in side effects between 2 groups, which supported the superior efficacy of standard dose relative to low dose.^[13]^ Another trial comparing standard (81 mg) with 3-fold reduced (27 mg) dose reported that the standard dose was significantly more effective against recurrences (*P* = .0151) and progression (*P* = .048) than the reduced dose in patients with multifocal tumors, and thus recommended continuing to use the standard dose for high-risk tumors.^[14]^ Other trials comparing 2 dose^[15,16]^ or 3 dose BCG group^[17,18]^ described that low-dose BCG showed a similar efficacy on recurrence or progression, and its toxicity was significantly lower compared with standard dose. A recent meta-analysis pooling 8 RCTs comparing BCG dose demonstrated that compared with standard BCG dose, low-dose BCG was not inferior to reduce the risk of tumor recurrence (HR, 1.15; 95% CI, 1.00–1.31; *P* = .05) and showed no significant difference in progression (HR, 1.08; 95% CI, 0.83–1.42; *P* = .57). Additionally, the use of low-dose BCG was significantly associated with lower incidence of severe (RR, 0.52; 95% CI, 0.36–0.74; *P* = .0003) and systemic side effects (HR, 0.57; 95% CI, 0.34–0.97; *P* = .01).^[40]^

RCTs regarding BCG duration have mainly focused on the evaluation of the efficacy of maintenance therapy compared to induction therapy only. One trial conducted by the Southwest Oncology Group demonstrated the significant impact of maintenance therapy relative to control (induction only). Patients randomized in the maintenance arm received a 6-week induction course followed by 3 weekly instillations at 3 and 6 months and every 6 months thereafter for 3 years (Southwest Oncology Group regimen) and showed no toxicities above grade 3. Estimated median RFS was 76.8 months in the maintenance arm and 35.7 months in the control arm (*P* = .0001) and 5-year OS was 78% in the control arm and 83% in the maintenance arm.^[22]^ This preventive impact of BCG maintenance therapy on the recurrence following TURBT was also identified in 2 other RCTs.^[24,25]^ In contrast, recent RCTs have described the insignificant effect of maintenance therapy in terms of the prevention of recurrence or progression.^[26,27]^ The Spanish Urological Club for Oncological Treatment 98013 study compared the recurrence and progression rates between BCG induction once-weekly for 6 weeks (no maintenance arm) and BCG induction followed by 1 BCG instillation every 3 months for 3 year (maintenance arm). Maintenance therapy had no significant advantages on the 5-year recurrence (33.5% in maintenance arm vs 38.5% in no maintenance arm) and progression rates (16.5% in maintenance arm vs 19.5% in no maintenance arm).^[26]^

Two RCTs provided conflicting results concerning the comparison of BCG strains. One RCT reported that there were no significant differences in RFS and adverse events between BCG Connaught and Tokyo strains.^[29]^ Another recent RCT demonstrated that, compared with BCG Tice, the BCG Connaught strain was significantly associated with greater 5-year RFS (74% in Connaught vs 48% in Tice; *P* = .0108).^[30]^

We tried to investigate the effective BCG strategies though a systematic review and meta-analysis for the previously reported RCTs. To the best of our knowledge, this study is the first meta-analysis evaluating the differences of the clinical outcomes according to the dose, duration, and strain of BCG. Standard dose and maintenance BCG therapy showed significant benefits in terms of reduction of recurrence risk following TURBT. These findings are partially consistent with the results of the previous trials,^[13,14,22,24,25]^ On the other hand, other clinical outcomes (PFS, CSS, and OS) were not significantly different depending on the dose and duration of BCG. Although previous meta-analysis on the BCG dose, which included many of the same studies observed in our analysis, concluded low-dose BCG was not inferior to standard dose BCG for reducing the risk of recurrence, the pooled HR for recurrence was marginal in light of 95% CI (1.00–1.31) and *P*-value (.05).^[40]^ Therefore, we interpreted the result of previous meta-analysis supported the superiority of standard dose BCG rather than noninferiority of low-dose BCG in terms of the prevention of recurrence, which consequently corresponds well with the results of the present study. The BCG strains could not be meta-analyzed because there have been too few studies; no meaningful conclusion on the effective BCG strain could be drawn from this study.

Several limitations should be considered for the interpretation of the present findings. First, in spite of the interstudy differences on the definition for the dose or duration of used BCG regimens in the included trials, we simply compared the clinical outcomes between binary variables (low vs standard dose, nonmaintenance vs maintenance) without head-to-head comparisons among diverse BCG regimens. Thus, we cannot draw a definite conclusion concerning the optimal BCG dose and duration. Some trials^[12,17,18]^ defined 120 mg as a standard dose and half or one/two-third of 120 mg as a low dose, while other trials^[13–16]^ used 80 or 81 mg as a standard dose and half or one/two-third of 80 or 81 mg as a low dose. For BCG duration, various definitions were also applied in terms of the maintenance duration. These nonunified definitions of BCG dose or duration in each trial may diversely affect the prognosis of NMIBC patients treated with TURBT. Second, unknown or uncontrolled variables that could not be clearly identified in the included trials might have affected the results of this analysis. Interinstitutional variation of TURBT techniques (ie, muscle layer resection, restaging TURBT), primary tumor size, and preoperative positive urine cytology, which were suggested as the important prognostic factors of NMIBC in previous studies,^[41–45]^ could not be adjusted through a multivariable analysis along with BCG. Third, the results of this systematic review and meta-analysis were based on unadjusted estimates, because some studies did not provide detailed information (Table 1). Finally, we cannot exclude the possibility of language bias by only including the articles published in English,^[46]^ despite no definite evidence of publication bias.

## 5 Conclusions

The current meta-analysis results indicate that in patients with NMIBC, the maintenance intravesical BCG strategies using standard dose may be effective to reduce recurrence risk after TURBT. However, the optimal dose, duration, and strain of BCG could not be definitely determined. Large scale, well-designed, and prospective studies, with stratification of the patients into risk group at randomization, will be required to establish the optimal guideline of BCG use to improve clinical outcomes in NMIBC.

## Supplementary Material

Supplemental Digital Content

## References

[1] Babjuk M Burger M Zigeuner R EAU Guidelines on non–muscle-invasive urothelial carcinoma of the bladder: update 2013. Eur Urol 2013;64:639–53. 23827737 10.1016/j.eururo.2013.06.003

[2] Power NE Izawa J Comparison of guidelines on non-muscle invasive bladder cancer (EAU, CUA, AUA, NCCN, NICE). Bladder Cancer 2016;2:27–36. 27376122 10.3233/BLC-150034 PMC4927900

[3] Borkowska EM Jedrzejczyk A Marks P EORTC risk tables – their usefulness in the assessment of recurrence and progression risk in non-muscle-invasive bladder cancer in Polish patients. Cent European J Urol 2013;66:14–20. 10.5173/ceju.2013.01.art5 PMC3921849 24578979

[4] Walczak R Bar K Walczak J The value of EORTC risk tables in evaluating recurrent non-muscle-invasive bladder cancer in everyday practice. Cent European J Urol 2014;66:418–22. 10.5173/ceju.2013.04.art6 PMC3992449 24757531

[5] Kapoor R Vijjan V Singh P Bacillus Calmette-Guerin in the management of superficial bladder cancer. Indian J Urol 2008;24:72–6. 19468364 10.4103/0970-1591.38608 PMC2684253

[6] Sylvester RJ van der Meijden APM Lamm DL Intravesical bacillus Calmette-Guerin reduces the risk of progression in patients with superficial bladder cancer: a meta-analysis of the published results of randomized clinical trials. J Urol 2002;168:1964–70. 12394686 10.1016/S0022-5347(05)64273-5

[7] Han RF Pan JG Can intravesical bacillus Calmette-Guérin reduce recurrence in patients with superficial bladder cancer? a meta-analysis of randomized trials. Urology 2006;67:1216–23. 16765182 10.1016/j.urology.2005.12.014

[8] Gårdmark T Jahnson S Wahlquist R Analysis of progression and survival after 10 years of a randomized prospective study comparing mitomycin-C and bacillus Calmette-Guérin in patients with high-risk bladder cancer. BJU Int 2007;99:817–20. 17244282 10.1111/j.1464-410X.2006.06706.x

[9] Duchek M Johansson R Jahnson S Bacillus Calmette-Guérin is superior to a combination of epirubicin and interferon-(2b in the intravesical treatment of patients with stage T1 urinary bladder cancer. a prospective, randomized, Nordic study. Eur Urol 2010;57:25–31. 19819617 10.1016/j.eururo.2009.09.038

[10] Sylvester RJ Brausi MA Kirkels WJ Long-term efficacy results of EORTC genito-urinary group randomized phase 3 study 30911 comparing intravesical instillations of epirubicin, bacillus Calmette-Guérin, and bacillus Calmette-Guérin plus isoniazid in patients with intermediate- and high-risk stage Ta T1 urothelial carcinoma of the bladder. Eur Urol 2010;57:766–73. 20034729 10.1016/j.eururo.2009.12.024 PMC2889174

[11] Burger M Oosterlinck W Konety B ICUD-EAU international consultation on bladder cancer 2012: non-muscle-invasive urothelial carcinoma of the bladder. Eur Urol 2013;63:36–44. 22981672 10.1016/j.eururo.2012.08.061

[12] Morales A Nickel JC Wilson JW Dose-response of bacillus Calmette-Guerin in the treatment of superficial bladder cancer. J Urol 1992;147:1256–8. 1569662 10.1016/s0022-5347(17)37532-8

[13] Yalçinkaya F Kamiş L Özteke O Prospective randomized comparison of intravesical BCG therapy with standard dose versus low doses in superficial bladder cancer. Int Urol Nephrol 1998;30:41–4. 9569110 10.1007/BF02550276

[14] Martínez-Piñeiro JA Flores N Isorna S Long-term follow-up of a randomized prospective trial comparing a standard 81 mg dose of intravesical bacille Calmette-Guérin with a reduced dose of 27 mg in superficial bladder cancer. BJU Int 2002;89:671–80. 11966623 10.1046/j.1464-410x.2002.02722.x

[15] Irie A Uchida T Yamashita H Sufficient prophylactic efficacy with minor adverse effects by intravesical instillation of low-dose bacillus Calmette-Guérin for superficial bladder cancer recurrence. Int J Urol 2003;10:183–9. 12657096 10.1046/j.0919-8172.2003.00607.x

[16] Martínez-Piñeiro JA Martínez-Piñeiro L Solsona E Has a 3-fold decreased dose of bacillus Calmette-Guerin the same efficacy against recurrences and progression of T1G3 and Tis bladder tumors than the standard dose? Results of a prospective randomized trial. J Urol 2005;174:1242–7. 16145378 10.1097/01.ju.0000173919.28835.aa

[17] Vijjan V Mandhani A Kappor R A randomized trial comparing low dose (40 or 80 mg) with standard dose (120 mg) of bacillus Calmette-Guerin for superficial bladder cancer. Indian J Urol 2006;22:317–21.

[18] Agrawal MS Agrawal M Bansal S The safety and efficacy of different doses of bacillus Calmette Guérin in superficial bladder transitional cell carcinoma. Urology 2007;70:1075–8. 18158020 10.1016/j.urology.2007.07.017

[19] Oddens J Brausi M Sylvester R Final results of an EORTC-GU cancers group randomized study of maintenance bacillus Calmette-Guérin in intermediate- and high-risk Ta, T1 papillary carcinoma of the urinary bladder: one-third dose versus full dose and 1 year versus 3 years of maintenance. Eur Urol 2013;63:462–72. 23141049 10.1016/j.eururo.2012.10.039

[20] Badalament RA Herr HW Wong GY A prospective randomized trial of maintenance versus non-maintenance intravesical bacillus Calmette-Guérin therapy of superficial bladder cancer. J Clin Oncol 1987;5:441–9. 3546618 10.1200/JCO.1987.5.3.441

[21] Gruenwald IE Stein A Rashcovitsky R A 12 versus 6-week course of bacillus Calmette-Guerin prophylaxis for the treatment of high risk superficial bladder cancer. J Urol 1997;157:487–91. 8996340

[22] Lamm DL Blumenstein BA Crissman JD Maintenance bacillus Calmette-Guérin immunotherapy for recurrence Ta, T1 and carcinoma in situ transitional cell carcinoma of the bladder: a randomized southwest oncology group study. J Urol 2000;163:1124–9. 10737480

[23] Palou J Laguna P MillÁN-RodrÍGuez F Control group and maintenance treatment with bacillus Calmette-Guérin for carcinoma in situ and/or high grade bladder tumors. J Urol 2001;165:1488–91. 11342902

[24] Koga H Ozono S Tsushima T Maintenance intravesical bacillus Calmette-Guérin instillation for Ta, T1 cancer and carcinoma in situ of the bladder: randomized controlled trial by the BCG Tokyo Strain Study Group. Int J Urol 2010;17:759–66. 20604814 10.1111/j.1442-2042.2010.02584.x

[25] Hinotsu S Akaza H Naito S Maintenance therapy with bacillus Calmette-Guérin Connaught strain clearly prolongs recurrence-free survival following transurethral resection of bladder tumour for non-muscle-invasive bladder cancer. BJU Int 2011;108:187–95. 21176079 10.1111/j.1464-410X.2010.09891.x

[26] Martínez-Piñeiro L Portillo JA Fernández JM Maintenance therapy with 3-monthly bacillus Calmette-Guérin for 3 years is not superior to standard induction therapy in high-risk non–muscle-invasive urothelial bladder carcinoma: final results of randomised CUETO Study 98013. Eur Urol 2015;68:256–62. 25794457 10.1016/j.eururo.2015.02.040

[27] Nakai Y Anai S Tanaka N Insignificant role of bacillus Calmette–Guérin maintenance therapy after complete transurethral resection of bladder tumor for intermediate- and high-risk non-muscle-invasive bladder cancer: results from a randomized trial. Int J Urol 2016;23:854–60. 27416975 10.1111/iju.13167

[28] Witjes WP Witjes JA Oosterhof GO Update on the Dutch Cooperative Trial: mitomycin versus bacillus Calmette-Guerin-Tice versus bacillus Calmette-Guerin RIVM in the treatment of patients with pTA-pT1 papillary carcinoma and carcinoma in situ of the urinary bladder. Dutch South East Cooperative Urological Group. Semin Urol Oncol 1996;14:10–6. 8727805

[29] Sengiku A Ito M Miyazaki Y A prospective comparative study of intravesical bacillus Calmette-Guérin therapy with the Tokyo or Connaught strain for non-muscle invasive bladder cancer. J Urol 2013;190:50–4. 23376145 10.1016/j.juro.2013.01.084

[30] Rentsch CA Birkhäuser FD Biot C Bacillus Calmette-Guérin strain differences have an impact on clinical outcome in bladder cancer immunotherapy. Eur Urol 2014;66:677–88. 24674149 10.1016/j.eururo.2014.02.061

[31] Moher D Liberati A Tetzlaff J Preferred reporting items for systematic reviews and meta-analyses: the PRISMA statement. BMJ 2009;339:b2535. 19622551 10.1136/bmj.b2535 PMC2714657

[32] McShane LM Altman DG Sauerbrei W Reporting recommendations for tumor marker prognostic studies. J Clin Oncol 2005;23:9067–72. 16172462 10.1200/JCO.2004.01.0454

[33] DerSimonian R Laird N Meta-analysis in clinical trials. Control Clin Trials 1986;7:177–88. 3802833 10.1016/0197-2456(86)90046-2

[34] Parmar MK Torri V Stewart L Extracting summary statistics to perform meta-analyses of the published literature for survival endpoints. Stat Med 1998;17:2815–34. 9921604 10.1002/(sici)1097-0258(19981230)17:24<2815::aid-sim110>3.0.co;2-8

[35] Tierney JF Stewart LA Ghersi D Practical methods for incorporating summary time-to-event data into meta-analysis. Trials 2007;8:16. 17555582 10.1186/1745-6215-8-16 PMC1920534

[36] Higgins JP Thompson SG Deeks JJ Measuring inconsistency in meta-analyses. BMJ 2003;327:557–60. 12958120 10.1136/bmj.327.7414.557 PMC192859

[37] Fernandez-Gomez J Madero R Solsona E Predicting nonmuscle invasive bladder cancer recurrence and progression in patients treated with bacillus Calmette-Guerin: the CUETO scoring model. J Urol 2009;182:2195–203. 19758621 10.1016/j.juro.2009.07.016

[38] Choi SY Ryu JH Chang IH Predicting recurrence and progression of non-muscle-invasive bladder cancer in Korean patients: a comparison of the EORTC and CUETO models. Korean J Urol 2014;55:643–9. 25324946 10.4111/kju.2014.55.10.643 PMC4198762

[39] Seo KW Kim BH Park CH The efficacy of the EORTC scoring system and risk tables for the prediction of recurrence and progression of non-muscle-invasive bladder cancer after intravesical bacillus Calmette-Guerin instillation. Korean J Urol 2010;51:165–70. 20414391 10.4111/kju.2010.51.3.165 PMC2855454

[40] Zeng S Yu X Ma C Low-dose versus standard dose of bacillus Calmette-Guerin in the treatment of nonmuscle invasive bladder cancer: a systematic review and meta-analysis. Medicine 2015;94:e2176. 26656345 10.1097/MD.0000000000002176 PMC5008490

[41] Koga F Kobayashi S Fujii Y Significance of positive urine cytology on progression and cancer-specific mortality of non–muscle-invasive bladder cancer. Clin Genitourin Cancer 2014;12:e87–93. 24169493 10.1016/j.clgc.2013.07.007

[42] Sfakianos JP Kim PH Hakimi AA The effect of restaging transurethral resection on recurrence and progression rates in patients with non-muscle invasive bladder cancer treated with intravesical bacillus Calmette-Guérin. J Urol 2014;191:341–5. 23973518 10.1016/j.juro.2013.08.022 PMC4157345

[43] Shindo T Masumori N Kitamura H Clinical significance of definite muscle layer in TUR specimen for evaluating progression rate in T1G3 bladder cancer: multicenter retrospective study by the Sapporo Medical University Urologic Oncology Consortium (SUOC). World J Urol 2014;32:1281–5. 24190368 10.1007/s00345-013-1205-1

[44] Zachos I Tzortzis V Mitrakas L Tumor size and T stage correlate independently with recurrence and progression in high-risk non-muscle-invasive bladder cancer patients treated with adjuvant BCG. Tumor Biol 2014;35:4185–9. 10.1007/s13277-013-1547-8 24375197

[45] Kim HS Ku JH Kim SJ Prognostic factors for recurrence and progression in Korean non-muscle-invasive bladder cancer patients: a retrospective, multi-institutional study. Yonsei Med J 2016;57:855–64. 27189277 10.3349/ymj.2016.57.4.855 PMC4951460

[46] Egger M Zellweger-Zähner T Schneider M Language bias in randomised controlled trials published in English and German. Lancet 1997;350:326–9. 9251637 10.1016/S0140-6736(97)02419-7

