# Supplementary material for: Dose, duration and strain of bacillus Calmette–Guerin in the treatment of nonmuscle invasive bladder cancer: Meta-analysis of randomized clinical trials
Source: Medicine (Baltimore). 2017 Oct 20;96(42):e8300. doi: 10.1097/MD.0000000000008300 (PMC5662397; doi:10.1097/MD.0000000000008300)
Supplement: Supplemental Digital Content [file medi-96-e8300-s001.doc]

Supplementary Figure legends

Fig. S1. **Funnel plots for publication bias test of prognosis in BCG dose. Each point represents a separate study for the indicated association. Vertical line represents the mean effects size. (A) recurrence-free survival, (B) progression-free survival, (C) cancer-specific survival, (D) overall survival.**


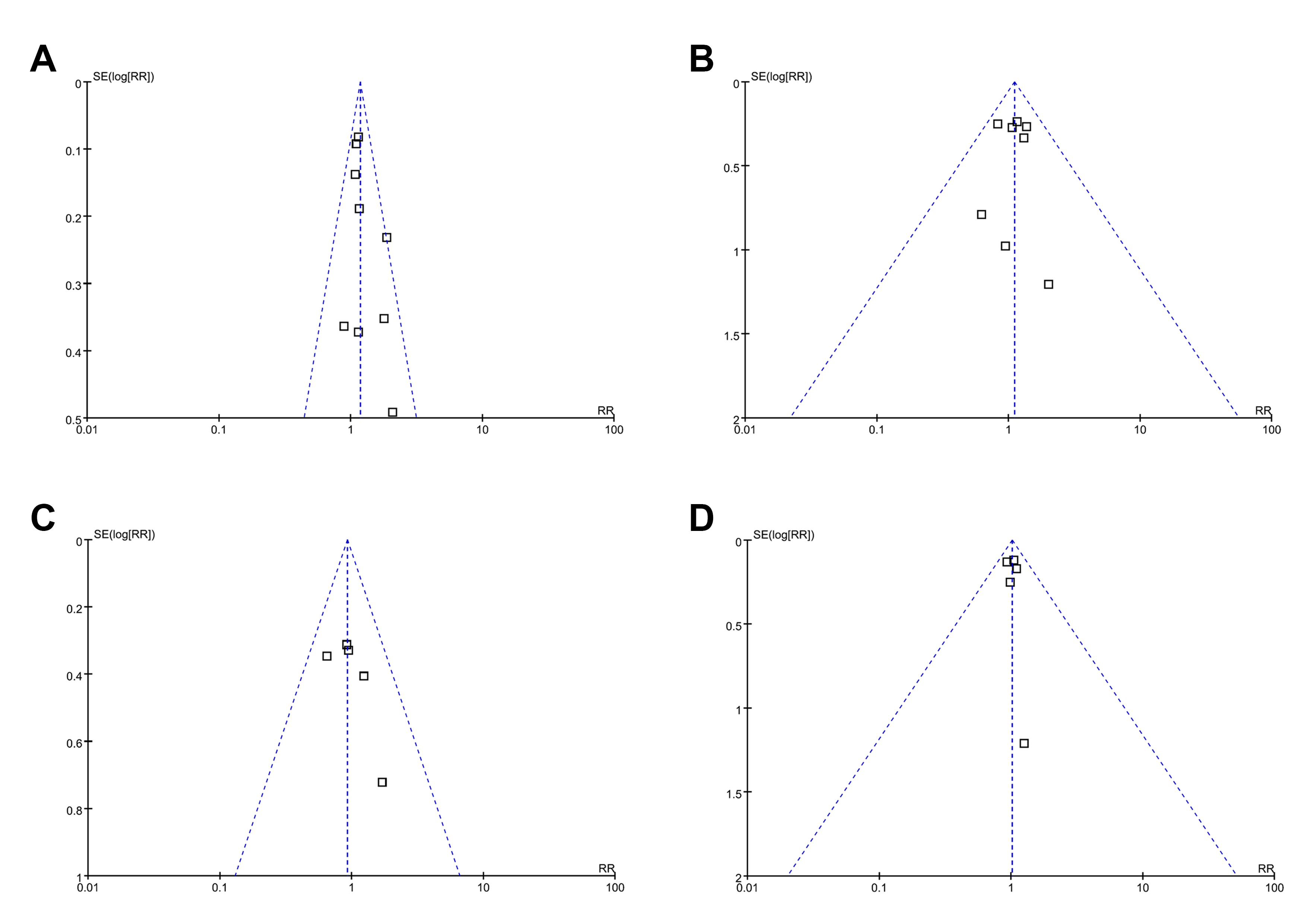


**Fig. S2.** Funnel plots for publication bias test of prognosis in BCG duration. Each point represents a separate study for the indicated association. Vertical line represents the mean effects size. (A) recurrence-free survival, (B) progression-free survival, (C) cancer-specific survival, (D) overall survival.


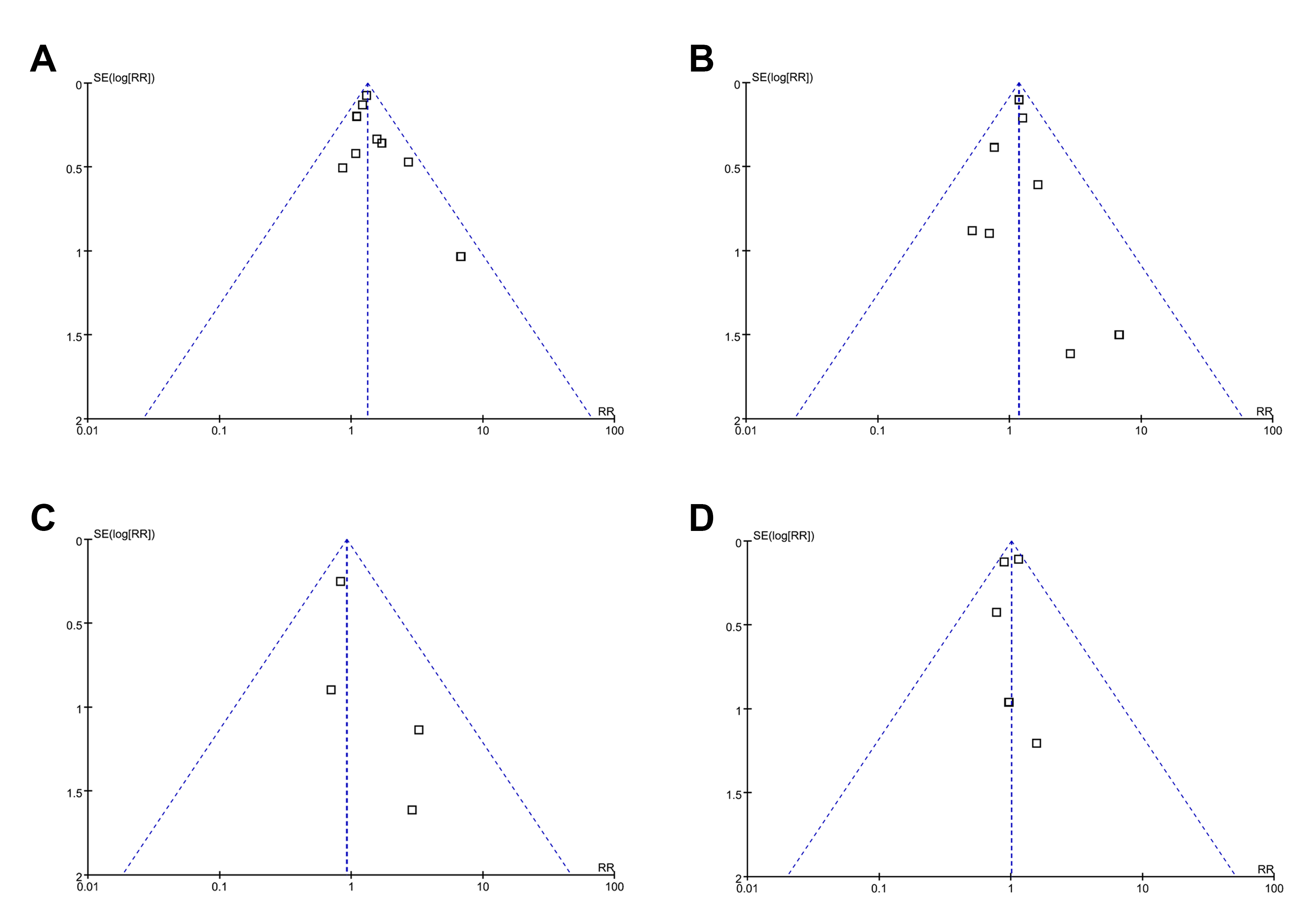


**Table S1.** Relative risk according to the strain of bacillus Calmette-Guerin

|  |  |  | Strain A |  | Strain B |  |  |
| --- | --- | --- | --- | --- | --- | --- | --- |
|  | Strain A | Strain B | No. events | No. total | No. events | No. total | RR (95% CI) |
| **Recurrence** |  |  |  |  |  |  |  |
| Witjes ^[28]^ | OncoTice | RIVM | 75 | 140 | 62 | 149 | 1.29 (1.01-1.64) |
| Sengiku ^[29]^ | Tokyo | Connaught | 42 | 86 | 45 | 92 | 1.00 (0.74-1.35) |
| Rentsch ^[30]^ | OncoTice | Connaught | 31 | 60 | 18 | 71 | 2.04 (1.28-3.25) |
| **Progression** |  |  |  |  |  |  |  |
| Witjes ^[28]^ | OncoTice | RIVM | 7 | 140 | 8 | 149 | 0.93 (0.35-2.50) |
| Rentsch ^[30]^ | OncoTice | Connaught | 7 | 60 | 4 | 71 | 2.07 (0.64-6.74) |
| **Cancer-specific mortality** |  |  |  |  |  |  |  |
| Rentsch ^[30]^ | OncoTice | Connaught | 0 | 60 | 5 | 71 | 0.11 (0.01-1.90) |
| **All-cause mortality** |  |  |  |  |  |  |  |
| Rentsch ^[30]^ | OncoTice | Connaught | 4 | 60 | 11 | 71 | 0.05 (0.003-0.85) |
